# Identification of learning-induced changes in protein networks in the hippocampi of a mouse model of Alzheimer's disease

**DOI:** 10.1038/tp.2016.114

**Published:** 2016-07-05

**Authors:** E Ferreira, D M Shaw, S Oddo

**Affiliations:** 1 Neurodegenerative Disease Research Center, Biodesign Institute School of Life Sciences, Arizona State University, Tempe, AZ, USA; 2 School of Life Sciences, Arizona State University, Tempe, AZ, USA

## Abstract

Memory loss is the most profound clinical manifestation in Alzheimer's disease (AD); however, the molecular mechanisms underlying these deficits are poorly understood. Identification of the molecular pathways involved in the onset of cognitive deficits may lead to the identification of key events in the pathogenesis of AD. Using isobaric tags for relative and absolute quantitation (iTRAQ) and proteomic methods, here we identified learning-induced changes in the hippocampal proteome of non-transgenic (NonTg) and 3 × Tg-AD mice, a widely used animal model of AD. We found that expression of 192 proteins was differentially regulated by learning in NonTg mice. Notably, of these 192 proteins, only 28 were also differentially regulated by learning in 3 × Tg-AD mice, whereas the levels of 164 proteins were uniquely changed in NonTg mice but not in 3 × Tg-AD mice. These data suggest that during learning, 3 × Tg-AD mice fail to differentially regulate 164 proteins. Gene ontology and protein interaction analyses indicated that these proteins were overrepresented in RNA processing, specifically RNA transport, splicing and mRNA translation initiation pathways. These findings suggest that mRNA-processing events that take place during learning and memory are significantly altered in 3 × Tg-AD mice.

## Introduction

Alzheimer's disease (AD) is a progressive neurodegenerative disorder and the number one cause of dementia in the United States. To this end, it is estimated that >13 million people will have this disease in the United States by 2050.^1^ Histopathologically, AD is characterized in part by the presence of extracellular neuritic plaques formed by Aß_40_ and Aß_42_ peptides. These peptides are generated as the result of sequential cleavage of the amyloid precursor protein (APP) through the amyloidogenic processing pathway.^2^ The other hallmark lesion of AD is the accumulation of intracellular neurofibrillary tangles consisting of hyperphosphorylated tau.^3^ These lesions often develop early in AD pathogenesis in the medial temporal lobe structures of the entorhinal cortex and hippocampus.^4^ One of the earliest clinical manifestations of AD is represented by impairments in memory formation, and as the disease progresses other cognitive domains become impaired, ultimately leading to a bedridden AD patient.^5^ To date, the precise mechanisms underlying the decline of learning and memory associated with AD are not known.

Immediate early genes (IEGs) play a key role in memory formation and consolidation.^6^ Most IEGs encode transcription factors that regulate the expression of other genes involved in the establishment of long-term memories.^7^ Growing evidence suggests that the alterations in these mechanisms may be an early event associated with AD pathogenesis. For example, cAMP-response element-binding protein-regulated transcription is impaired in multiple mouse models of AD, and restoring its function is sufficient to rescue cognitive deficits in 3 × Tg-AD mice, a widely used animal model of AD.^8, 9, 10^ In this work, we sought to identify learning-induced changes in protein levels that may be responsible for underlying cognitive deficits in AD.

The use of high-throughput quantitative proteomics has offered significant insights into furthering the understanding of AD pathogenesis at a global molecular level.^11, 12^ These studies have yielded significant results when comparing the proteome of the AD brain versus control cases. For example, the proteomic analysis of human samples compared to control cases has led to the identification of potential biomarkers that can aid in differentiating mild cognitive impairment from AD, as well as biomarkers with both diagnostic value and prognostic value regarding mild cognitive impairment to AD progression.^13, 14^ Proteomic studies in mice have led to important insights of AD pathogenesis as well, such as characterization of the proteomic changes in the hippocampus throughout AD pathogenesis when comparing non-transgenic (NonTg) to transgenic mice with AD-like phenotypes.^15^ The quantitative proteomic strategy of using isobaric tag for relative and absolute quantitation (iTRAQ) has been a particularly powerful proteomics tool. As an example, iTRAQ method has been applied to specific regions of the mouse brain (that is, hippocampus, parietal cortex, cerebellum) in normal versus AD-like mice to yield the identification of numerous proteins found to be differentially regulated in AD-like mice in a site-specific manner, with most of these proteins found to be involved in molecular transport, nervous system development, synaptic plasticity and apoptosis.^16^

While these studies have generated significant knowledge regarding proteomic changes due to AD pathogenesis, there is a clear lack of quantitative proteomic studies designed specifically to identify the learning-induced proteomic changes affected by AD. This is a critical distinction, because events in AD pathogenesis are linked to memory formation and consolidation.^17^ To better understand how AD pathogenesis impedes memory, we employed iTRAQ to measure proteomic differences induced by Morris water maze (MWM) training in 3 × Tg-AD and NonTg mice. Differences in quantitative proteomic changes during MWM training between NonTg and 3 × Tg-AD groups were then used in conjunction with publicly available bioinformatics databases to determine which cellular processes and signaling pathways may account for the learning deficits associated with AD pathology. This led to the finding of several major pathways found to be significantly affected by 3 × Tg-AD pathology during learning and memory training.

## Materials and methods

### Mice

The generation of the 3 × Tg-AD mice has been described previously.^10^ All mice were housed four to five to cage at 23 ºC, kept on a 12 h light/dark cycle and were given *ad libitum* access to food and water. In our colony of 3 × Tg-AD mice, males show a large neuropathological variability, even between littermates. In contrast, female 3 × Tg-AD mice do not show such large variability and their phenotype changes as a function of age in a predictable manner. Therefore, only female mice were used for the experiments described here. All animal procedures were approved by The Institutional Animal Care and Use Committee of Arizona State University.

### Protein extraction from mouse hippocampus for iTRAQ labeling

Mice were killed by CO_2_ asphyxiation 2 h after final MWM training. Their brains were removed and sagittally bisected. The left hippocampus was removed and used for iTRAQ labeling; the right hippocampus was removed and stored at −80 °C until use for western blot analysis. Hippocampi were homogenized and lysed in a lysis buffer containing 8 m urea, 50 mm HEPES, pH 8.5. After centrifugation at 20 000*g* and 4 °C for 60 min, the protein concentration of the supernatant was determined using BCA assay (Thermo Scientific, San Jose, CA, USA). The protein disulfide bonds in the supernatant were reduced for 40 min with 5 mm dithiothreitol at room temperature and alkylated for 40 min with 15 mm iodoacetamide in the dark. Alkylated protein samples were diluted with 100 mm HEPES pH 8.5 to 2 m urea followed by digestion overnight at 37 °C with trypsin in a 1:50 enzyme-to-substrate ratio (Promega, Madison, WI, USA, V5113). After digestion, the peptide mixtures were acidified with trifluoroacetic acid (TFA) to 1%, and subjected to C18 solid-phase extraction (Sep-Pak, Waters, Milford, MA, USA). Finally, the desalted peptide samples were dried in a vacuum concentrator and stored at −20 °C for peptide TMT labeling.

### iTRAQ protocol

The desalted peptides were dissolved in 100 μl of 100 mm TEAB, pH 8.5. The peptide concentration was measured using a MicroBCA assay (Thermo Scientific), and 100 μg of digested peptides for the samples were incubated with 40 μl TMT reagents (Thermo Scientific) for 1 h at room temperature. Reactions were quenched by adding 8 μl of 5% hydroxylamine and incubating for 15 min. TMT-labeled samples (S1, S5, S9, S13, S17, S21) were combined at a 1:1:1:1:1:1 ratio, desalted and dissolved in an isoelectric focusing (IEF) buffer consisting of 5% glycerol and 2% IPG buffer (pH 3–10, GE Healthcare, Arlington Heights, IL, USA). The peptide mixtures were loaded into 24 wells over a 24-cm Immobiline Dry Strip, pH 3–10 (GE Healthcare), and separated on a 3100 OFFGEL Fractionator (Agilent Technologies, Santa Clara, CA, USA) according to the manufacturer's instructions. A total of 24 peptide fractions were obtained, acidified using 1% TFA and desalted prior to liquid chromatography–tandem mass spectrometry (LC-MS/MS) analysis. The dried peptides were dissolved in 20 μl of 0.2% formic acid and subjected to nanoLC-MS/MS analysis. The peptides were separated with the homemade C18 reverse-phase column packed with 15 cm of ReproSil-Pur C18-AQ resin (3 μm, 120 Å, Dr Maisch, Ammerbuch, Germany). Peptides were eluted with a 2 h gradient of 6–30% acetonitrile in 0.1% formic acid at a flow rate of 300 nl min^−1^ using an Easy nLC 1000 system (Thermo Scientific). The eluted peptides were analyzed directly with a Q Exactive (Thermo Fisher Scientific, Anthem, AZ, USA). The spray voltage was set to 2.0 kV. Full-scan MS survey spectra (*m/z* 300–1600) in profile mode were acquired in the Orbitrap with a resolution of 30 000 after accumulation of 1 000 000 ions, followed by 4 CID fragmentation (collision-induced dissociation; normalized collision energy, 35% activation time 30 ms, isolation width 1.0  *m/z*) and 4 HCD fragmentation (higher-energy collisional dissociation, normalized collision energy 70% maximum inject time 300 ms; mass resolution 7500; activation time 30 ms) for the 4 most intense peptide ions selected from the survey scan in the Orbitrap.

### Data processing, statistical analysis and bioinformatics

Raw MS data files were processed with Proteome Discoverer (Version 1.4, Thermo Fisher Scientific) and searched against Uniprot mouse protein sequence database. The parameters were set as follows: fixed modifications, carbamidomethylation (C), TMT/+229D-K and TMT/+229D-N terminal; oxidation (M) (variable); the enzyme specificity was set to trypsin; the maximum missed cleavages were set to 2; the precursor ion mass tolerance was set to 10 p.p.m. while MS/MS tolerance was 0.5 Da. The false positive rate was set as 0.01. A TMT 6-plex quantitation method in Proteome Discoverer was applied for HCD-based peptide quantitation. This allowed for quantitative measurement of each detected protein as relative abundance ratios. Ratios were used to determine 1.2-fold change levels and significance of these fold-changes (*P<*0.05) using an online distribution-free permutation-based quantitative proteomics *P*-value calculator (http://qppc.di.uq.edu.au). To account for false discovery, Benjamini–Yekutieli method was used.

ConsensusPathDB-mouse (http://cpdb.molgen.mpg.de/MCPDB) is a bioinformatics web-tool that integrates interaction networks in *Mus musculus*, including binary and complex protein–protein, genetic, metabolic, signaling, gene regulatory and drug–target interactions, as well as biochemical pathways. Data originate from 16 public resources for interactions and interactions curated from the literature. Overrepresentation analysis was used to identify pathway-based sets in which the 164 proteins of interest were enriched.

STRING (Search Tool for the Retrieval of Interacting Genes/Proteins) is a database of known and predicted protein interactions. The interactions include direct (physical) and indirect (functional) associations; they are derived from four sources: genomic context, high-throughput experiments, coexpression and previous knowledge. Proteins of interest were mapped to the STRING10.0 *M. musculus* database to identify high-confidence protein–protein interaction networks using confidence analysis.

The PANTHER (Protein Analysis Through Evolutionary Relationships) Classification System was used to carry out gene ontology (GO) analysis. PANTHER is designed to classify proteins (and their genes) to facilitate high-throughput analysis. Proteins of interest were classified according to family and subfamily, molecular function and biological process. Detailed methods of how proteins are classified have been previously described.^18^

### Protein extraction and western blots

Frozen hippocampi were homogenized using a dounce homogenizer in T-PER buffer (Thermo Scientific) supplemented with a protease inhibitor cocktail tablet (Roche, Indianapolis, IN, USA) and phosphatase inhibitors. Samples were then centrifuged at 25 000*g* for 30 min at 4 °C. The supernatant was stored as the soluble fraction and used for western blot experiments for validation of iTRAQ data.

Proteins from the soluble fraction were loaded on precast SDS–polyacrylamide gel electrophoresis gels and run under reducing conditions, after which they were transferred to a nitrocellulose membrane. Membranes were then incubated in a 5% milk solution in T-TBS (0.1% Tween 20, 100 mm Tris, pH 7.5; 150 mm NaCl) 1 h at 25 °C, washed and incubated in primary antibody overnight at 4 °C. Membranes were washed in TBS-T for 30 min and incubated in goat anti-mouse IRDye 680LT (LI-COR Biotechnology, Lincoln, NE, USA) or goat anti-rabbit IRDye 800CW LI-COR secondary antibodies (LI-COR Biotechnology) (1:10 000) for 1 h at 25 °C. After final washes, membranes were imaged and analyzed using the LI-COR Odyssey (LI-COR Biotechnology). Protein densitometry was calculated by dividing the integrated intensity of the protein of interest by integrated intensity of beta-actin loading control.

### Antibodies

PLC-γ1, eIF4H, α-Parvin and ß-actin were obtained from Cell Signaling Technology (Danvers, MA, USA).

## Results

### Identification of learning-induced proteome changes

Learning-induced changes in protein expression are known to be at the basis of memory formation and consolidation.^19, 20, 21^ We sought to identify the learning-induced changes in protein expression between 3 × Tg-AD mice and NonTg mice. To this end, we used 12–15-month-old mice (*n*=4 per genotype). At this age, the 3 × Tg-AD mice have well-documented cognitive decline, associated with the build-up of amyloid-β deposits and neurofibrillary tangles (Supplementary Figure 1 and refs 10, 18, 22). Mice were trained in the spatial version of the MWM for 5 consecutive days, four training trials per day. Thirty minutes after the last training trials, mice were killed and their hippocampi quickly removed and frozen in dry ice. These mice that underwent learning will hereafter be referred to as 3 × Tg-AD-L and NonTg-L mice. Per each genotype, control mice were killed directly from their home cage and are hereafter referred to as Naive mice, or 3 × Tg-AD-N and NonTg-N. The left hippocampi were then processed for iTRAQ as described in Materials and methods section and Supplementary Figure 2.

**Figure 1:**
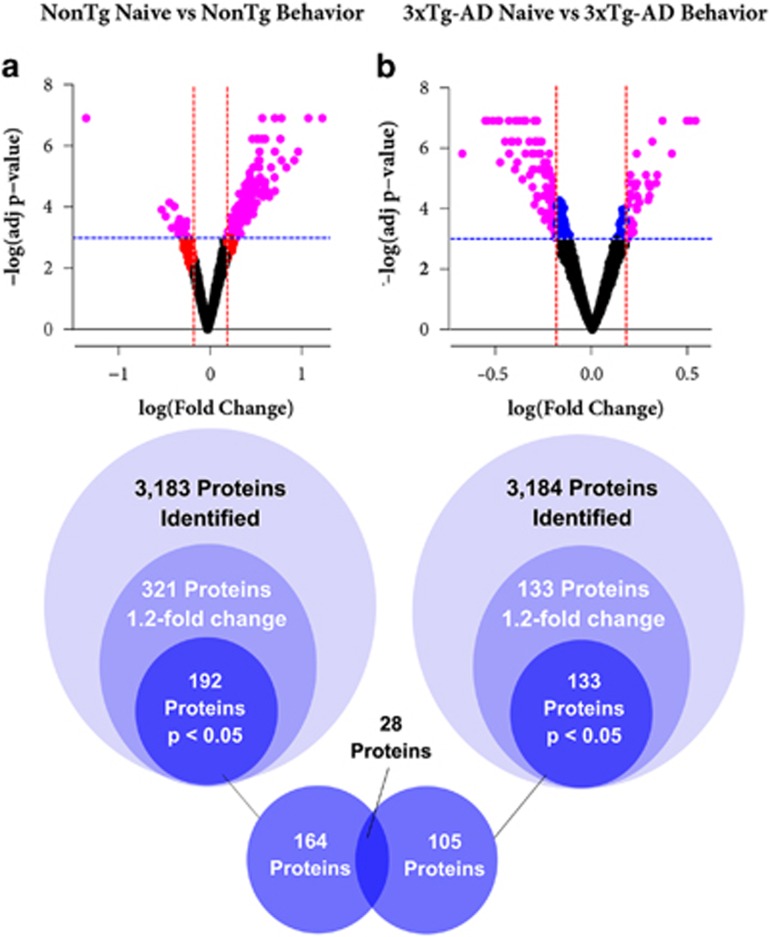
Representation of how iTRAQ data were processed and analyzed. We identified 3183 proteins that were differentially regulated in NonTg-N and NonTg-L groups (**a**) and 3184 proteins that were differentially regulated in 3 × Tg-AD-N and 3 × Tg-AD-L groups (**b**). Of these, 321 proteins met the first criterion and 192 met both criteria in the NonTg group. 133 proteins met the first criterion and all of them met the second criteria in the 3 × Tg-AD group. In the volcano plots, each dot represents a protein. In black are the proteins that did not meet either criterion. In blue are the proteins that met only the first criterion, *P<*0.05. In red are those proteins that met only the second criterion of a 1.2-fold change. In purple are those proteins that met both criteria. Proteins that met both criteria in both groups of mice were eliminated to determine which proteins are affected by learning events in NonTg hippocampi, but are not affected in 3 × Tg-AD hippocampi. This led to the identification of 164 proteins with learning-induced changes in the hippocampi of NonTg mice, but not in 3 × Tg-AD mice. AD, Alzheimer's disease; NonTg, non-transgenic.

We first compared NonTg-N mice with NonTg-L mice to identify changes in protein levels that are related to learning and memory. A total of 3183 proteins were identified and, of these, 321 satisfied the arbitrary cut-off criterion of a 1.2-fold change in levels between NonTg-L and NonTg-N. The 1.2-fold cut-off is a value routinely used in data analyses of iTRAQ data.^23, 24, 25^ Of the 321 proteins that met this first criterion, 192 had a *P*-value<0.05 (Figure 1). To determine whether the same proteins were also differently regulated by learning in 3 × Tg-AD mice, we repeated the same experiments comparing the steady-state levels of the hippocampal proteome between 3 × Tg-AD-N mice and 3 × Tg-AD-L mice. We detected 3184 different proteins in the hippocampi of 3 × Tg-AD mice. Of these, 133 satisfied the 1.2-fold change in proteins levels. All of these had adjusted *P*-values<0.05 between 3 × Tg-AD-N and 3 × Tg-AD-L mice (Figure 1). We postulated that some of the 192 proteins whose levels changed after learning in NonTg mice might be involved in memory formation and consolidation. Therefore, we asked which of these 192 proteins were not differentially expressed when compared 3 × Tg-AD-N and 3 × Tg-AD-L mice. We found that of these proteins, 28 were also found to have altered levels between 3 × Tg-AD-N and 3 × Tg-AD-L mice (Supplementary Table 1). This yielded a total of 164 unique proteins whose levels were altered following learning in the hippocampus of NonTg mice, and not in 3 × Tg-AD mice (Figure 1). The top 20 of these proteins are shown in Table 1 and the complete list is in the Online Supplementary Material. We also identified 105 unique proteins whose levels were altered following learning in the hippocampus of 3 × Tg-AD mice but not in NonTg mice. The complete list of these proteins is in the Online Supplementary Material. We hypothesize that some of the 164 proteins that are differentially expressed following learning in NonTg mice but not in 3 × Tg-AD mice may contribute to the memory deficits in 3 × Tg-AD mice.

### Functional classification of proteins regulated in hippocampus during learning and memory

**Figure 2:**
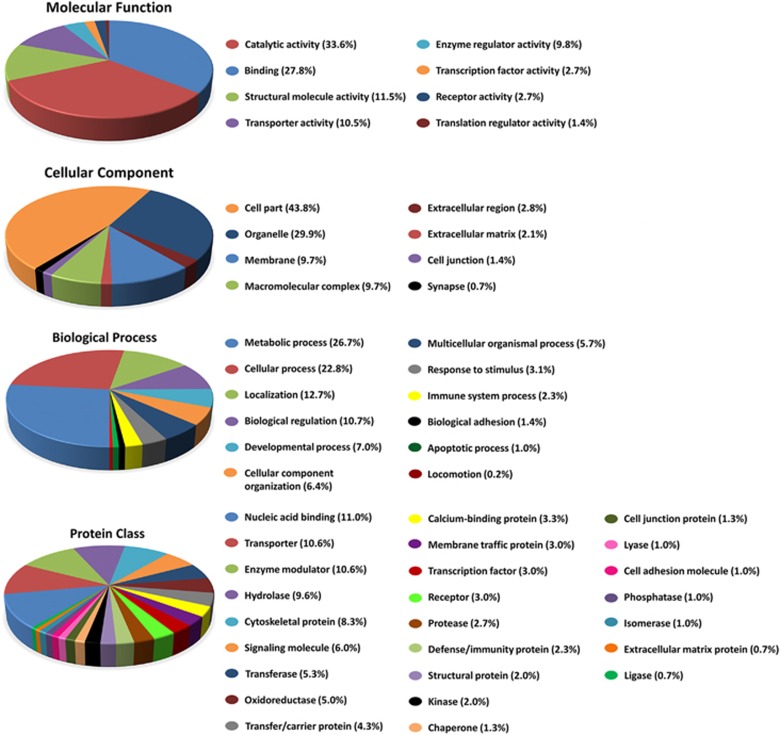
Pie chart depicting the functional classification of differentially regulated hippocampal proteins during learning and memory between NonTg and 3 × Tg-AD mice. The iTRAQ-identified changes in the hippocampal proteome were characterized using gene ontology (GO) analysis. Subcellular and functional categories were based on the annotations of GO using the PANTHER Classification System for the categories of Biological process, Molecular function, Protein class and Cellular component.

To gather insight into the biological functions of the 164 proteins found to have altered expression during learning in the hippocampi of NonTg mice but not in those of 3 × Tg-AD mice, we conducted GO analysis using the PANTHER classification system database. The PANTHER classification system classifies proteins and their respective genes, by molecular function, biological process, cellular component and protein class to facilitate high-throughput analysis of a large set of proteins/genes.^26, 27^ GO analysis of the 164 proteins of interest showed that these proteins represent numerous and diverse classes of proteins (Figure 2). The largest proportion of the protein class identified represents nucleic acid-binding proteins, suggesting that regulation of gene expression may be disproportionately affected during learning and memory in 3 × Tg-AD mice. Consistent with this observation, we also found that some of the 164 differentially regulated proteins are part of the transporter and enzyme modulator proteins, suggesting that the proteins with altered levels during spatial learning may have an important role in protein/RNA trafficking, as well as changes in metabolism. This is further supported when viewing our proteins of interest by their biological process classification, as the highest percentage of proteins are involved in metabolic processes. The next highest percentage of biological process classifications is cellular process, which includes regulation of cell cycle, cell growth, cell communication and cell proliferation, further indicating that these proteins regulated during learning and memory are disproportionately linked to processes involving growth. The classification of these proteins by cellular component indicates that the majority reside in organelles. Last, the classification of the 164 proteins of interest by their molecular function further corroborates the idea that the majority of these proteins are involved in metabolism, transport, regulation of growth and regulation of gene expression as the majority of them have catalytic activity function or binding activity (Figure 2). Taken together, these results indicate that the proteins significantly affected during learning by AD-like pathology are involved in gene expression, metabolism, cell growth and protein transport. The altered regulation of these protein classes would significantly impact the synthesis and regulation of proteins required for memory consolidation during a spatial learning training exercise such as MWM.^28^

### Pathway analysis of proteins regulated in hippocampus during learning and memory

**Figure 3:**
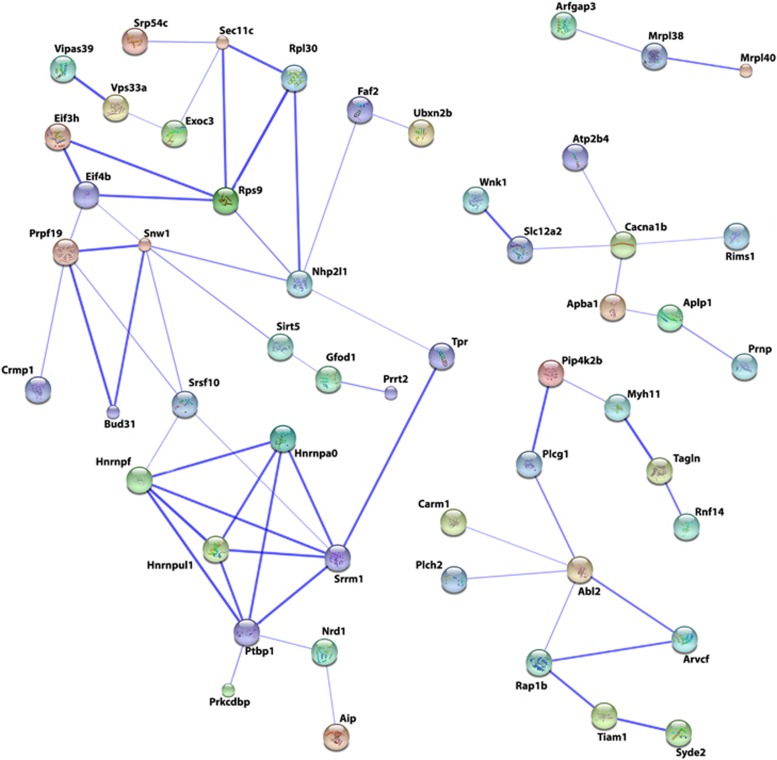
STRING v10.0 protein–protein interaction network based on input of 164 proteins of interest. Enrichment data were generated using the iTRAQ-identified network of learning-induced changes in proteins differentially expressed in the hippocampus between NonTg and 3 × Tg-AD mice during learning and memory. The database maps input proteins to enrich known pathways based on the following active prediction method criteria: interactions in other existing public databases, experimental data, gene fusion, neighborhood proteins and text mining. Colored nodes indicate input proteins. Stronger association of protein–protein interaction is represented by thicker lines connecting protein nodes. Node size is indicative of amount of protein structure information. Proteins within this interaction network that comprise a network of strong interactions and significantly enrich the major RNA splicing pathway (*P<*0.001) are Hnrnpa0, Hnrnpf, Hnrnpul1, Srrm1 and Ptbp1. Also within this interaction network are input proteins significantly enriched to RNA translation initiation (*P<*0.01). These proteins, in part, consist of Eif3h, Eif4b, Rps9 and Rpl30.

To determine the specific cellular pathways in which our proteins of interest play significant roles, we used the online proteomics tool Consensus Path Database (mouse) enrichment analysis function. This database integrates protein–protein interaction networks using data originating from 16 public resources.^29^ Enrichment analysis using the 164 proteins with learning-induced changes in protein levels in NonTg hippocampi resulted in the finding that these proteins are significantly enriched in RNA-processing pathways such as processing of intron-containing pre-mRNA (*P<*0.001), mRNA splicing (*P<*0.001) and mRNA processing (*P<*0.01), as well as gene expression and metabolism pathways (Table 2). To further identify the protein networks most affected during learning by AD-like pathology, we used the STRING v10. This web-based database and tool maps protein–protein interaction networks based on known and predicted protein–protein interactions.^30^ Figure 3 shows the protein networks resulting from STRING analysis confidence view of the 164 input proteins. The thicker lines represent stronger protein–protein associations. The largest and most prominent network includes several modules of high-confidence interactions, all falling under the umbrella of gene expression. For example, one cluster within this network contains a group of heterogeneous nuclear ribonucleoproteins (that is, hnrnpf, hnrnpul1, hnrnpa0) that function in pre-mRNA processing such as transport from the nucleus and spliceosome formation. Another module of high-confidence associations contained within the largest network, resulting from STRING analysis, is a group of proteins involved in initiation of protein translation, consisting of Eif3h, Eif4b, Rps9 and Rpl30. A different network includes several proteins involved in synaptic membrane regulation, such as proteins involved in ion-channel regulation (that is, Slc12a2, Wnk1) and synaptic vesicle exocytosis (Rims1). Another smaller network of note resulting from STRING analysis includes proteins involved in synthesis and regulation of phosphatidylinositol phosphates (that is, Pip4k2b, Plcg1). Last, the inclusion of three associated proteins (Arfgap3, Mrpl38 and Mrpl40) involved in mitochondrial protein synthesis suggests that learning-induced increases in metabolic needs may be affected by AD pathogenesis.

To validate the proteomics data obtained by iTRAQ, we measured learning-induced changes in three randomly chosen proteins, α-Parvin, eIF4H and PLC-γ1, by western blot in the hippocampi of NonTg-N and NonTg-L mice. We found that the steady-state levels of α-parvin and PLC-γ1 were significantly increased by learning in the hippocampi of NonTg mice (*P<*0.05), as detected by western blot. In contrast, the levels of eIF4H were not statistically significant between NonTg-N and NonTg-L (Supplementary Figure 3). These western blots data were consistent with changes detected with iTRAQ (gray bars in Supplementary Figure 3).

## Discussion

In an effort to identify key cellular networks most affected by AD pathogenesis during learning and memory, we employed a quantitative proteomic approach, iTRAQ, to find learning-induced changes in hippocampal protein level that are unique between NonTg and 3 × Tg-AD mice. The overexpression of the two transgenes in 3 × Tg-AD mice may be sufficient to alter the expression of proteins within the networks we identified in our analyses. However, the expression of proteins that are differentially regulated solely because of the tau and APP transgenes would not change between 3 × Tg-AD-N and 3 × Tg-AD-L. In other words, our analytical approach greatly reduces the identification of false positive hits driven solely by the presence of the transgenes in 3 × Tg-AD mice.

We identified 162 proteins that were differentially expressed after learning in NonTg mice but not in 3 × Tg-AD mice. We used them as input for GO analysis, as well as pathway overrepresentation analysis, to identify the key cellular processes and protein networks where these proteins of interest are enriched. Top hits for GO analysis revealed that learning-induced alterations to the hippocampal proteome are overrepresented by regulators of gene expression, metabolism, cell growth and protein transport. Consistent with GO analysis results, pathway analysis using protein interaction databases and bioinformatic web-tools showed that these proteins of interest were significantly enriched in regulation of gene expression networks such as mRNA translation initiate on, pre-mRNA transport and splicing, as well as pathways within metabolic networks such as mitochondrial protein synthesis and regulation of phosphatidyl inositols. Taken together, these results indicate that gene expression pathways, specifically regulation of pre-mRNA transport and splicing, as well as initiation of translation, play a prominent role in the learning and memory pathways affected by AD pathogenesis.

Of the GO and pathway analysis results reported above, the most prominent and overrepresented cellular networks affected by AD pathology involve a host of RNA-processing events, including formation of spliceosome with intron-containing pre-mRNA, transport of mRNA out of the nucleus and initiation of mRNA translation for protein synthesis. Consistent with our finding, strong published evidence indicates that alterations in protein translation may contribute to the pathogenesis of AD.^31^ We, and others, have previously shown that the mammalian target of rapamycin, a master regulator of protein translation,^32^ is upregulated in human AD brains.^18, 33, 34, 35^ These alterations in protein translation may lead to the changes of proteins involved in AD pathogenesis and learning and memory. To this end, previous studies have shown that the mRNA transcripts of IEGs involved in learning and memory consolidation, such as *Arc*, are regulated by localized transport at dendritic compartments of active synapses.^36^ This targeted sequestration of IEG mRNA transcripts at dendritic compartments allows for rapid downstream IEG expression in the nucleus of a neuron activated by behavioral events, such as MWM training.^37^ This makes the transport of mRNAs, as well as mRNA translation machinery, to dendritic compartments crucial for synaptic plasticity required for learning and memory consolidation. Together, our data suggest that the development of neuropathology in the hippocampi of 3 × Tg-AD mice may affect the learning-induced differential regulation of these RNA-processing pathways and thus impede IEG mRNA expression and downstream signaling cascades that ultimately cause deficits in learning.

In addition to events that affect mRNA transport and translation of initiation, RNA splicing is also a major pathway overrepresented within protein–protein networks of our proteins of interest. Indeed, a report of a proteomics study analyzing the insoluble proteome of human AD brains revealed a significant enrichment of RNA spliceosome proteins, in particular the U1 small nuclear ribonucleoprotein.^38^ Comparisons of RNA splice variants between human AD brains to control cases showed dysregulated RNA processing, including unspliced RNA species, within AD brains. This could potentially lead to accumulation of unspliced transcripts that are involved in cellular pathways key to memory consolidation.

The findings reported here point to several major protein networks significantly altered in the hippocampus due to the AD-like pathology exhibited by 3 × Tg-AD mice. These networks involving synaptic membrane ion channels, mitochondrial protein synthesis and most prominently RNA processing, transport and translation present avenues for future studies to further elucidate the precise molecular mechanisms responsible for learning deficits in AD pathogenesis. One opportunity for further investigation presented by these data are the investigation of the RNA profiles of 3 × Tg-AD mice during learning and memory events in comparison to NonTg mice. Specifically, examining splicing alterations within mRNA transcripts of genes involved in APP processing, tau phosphorylation, autophagy and proteasomal degradation pathways between NonTg and 3 × Tg-AD mice would be an important comparison to examine. Further study of synaptic membrane dynamics is also warranted, specifically looking at transport of IEG mRNA to dendritic compartments.

## Figures and Tables

**Table 1:** Top 20 proteins showing greatest learning-induced change in expression levels unique to NonTg mice

*UniProt ID*	*Gene symbol*	*Gene name*	*Normalized* P-*values*	*FC in NonTg-L relative to NonTG-N*	*Learning-induced regulation*
Q8BHB9	Clic6	Chloride intracellular channel protein 6	0.000999001	3.405049197	Up
A2AJI0	Map7d1	MAP7 domain-containing protein 1	0.002997003	2.614151998	Up
P02802	Mt1	Metallothionein-1	0.002997003	2.505045838	Up
Q9ER35	Fn3k	Fructosamine-3-kinase	0.006993007	2.301152791	Up
Q99J77	Nans	*N*-acetylneuraminic acid synthase	0.001998002	2.179955834	Up
Q9CQ10	Chmp3	Charged multivesicular body protein 3	0.007992008	2.175759902	Up
Q3UGX3	Nat8l	*N*-acetylaspartate synthetase	0.001998002	2.148748441	Up
Q8BQ30	Ppp1r18	Phostensin	0.005994006	2.028196723	Up
Q60673	Ptprn	Receptor-type tyrosine-protein phosphatase-like N	0.002997003	2.02741379	Up
Q5U3K5	Rabl6	Rab-like protein 6	0.002997003	2.025783874	Up
Q7TN79	Akap7	A-kinase anchor protein 7 isoform gamma	0.002997003	2.025783874	Up
Q8K2M0	Mrpl38	39 S ribosomal protein L38, mitochondrial	0.010989011	1.941987474	Up
Q3UJB9	Edc4	Enhancer of mRNA-decapping protein 4	0.011988012	1.905276998	Up
Q9ESW4	Agk	Acylglycerol kinase, mitochondrial	0.028971029	−1.621647401	Down
Q52KI8	Srrm1	Serine/arginine repetitive matrix protein 1	0.018981019	−1.559280245	Down
Q91WK2	Eif3h	Eukaryotic translation initiation factor 3 subunit H	0.026973027	−1.479532814	Down
Q8C0L9	Gpcpd1	Glycerophosphocholine phosphodiesterase GPCPD1	0.038961039	−1.431324152	Down
Q9WV52	Plek2	Pleckstrin-2	0.044955045	−1.37844914	Down
Q9JLV1	Bag3	BAG family molecular chaperone regulator 3	0.046953047	−1.31003492	Down

Abbreviation: NonTg, non-transgenic.

**Table 2:** Pathways enriched by proteins identified with learning-induced hippocampal level changes

*Pathway name*	*Pathway set size*	*Candidates contained*	P*-value*	*Pathway source*
Processing of capped intron-containing pre-mRNA	144	7 (4.9%)	3.38 × 10^−4^	Reactome
mRNA splicing	112	6 (5.4%)	5.46 × 10^−4^	Reactome
d-my-inositol-5 phosphate metabolism	19	3 (15.8%)	6.44 × 10^−4^	MouseCyc
mRNA processing	163	7 (4.3%)	7.12 × 10^−4^	Reactome
PIP metabolism	54	4 (7.4%)	1.46 × 10^−3^	MouseCyc
Gene expression	720	14 (2.0%)	4.52 × 10^−3^	Reactome
