# Supplementary material for: Identification of learning-induced changes in protein networks in the hippocampi of a mouse model of Alzheimer's disease
Source: Transl Psychiatry. 2016 Jul 5;6(7):e849–. doi: 10.1038/tp.2016.114 (PMC4969764; doi:10.1038/tp.2016.114)
Supplement: Supplementary Table 1 [file tp2016114x1.doc]

**Supplementary Table 1. List of the 28 proteins with learning-induced hippocampal changes in both NonTg and 3xTg-AD mice**

| **Gene Symbol** | **Mean Ratio**  **NonTg-L/NonTg-N** | **Mean Ratio**  **3xTg-AD-L/3xTg-AD-N** |
| --- | --- | --- |
| Alpha-parvin | 0.72248 | 0.72648 |
| Keratin, type II cytoskeletal | 0.26519 | 0.81593 |
| Isoform 3 of Apoptosis-inducing factor 3 | 1.40547 | 1.26248 |
| ATP synthase protein 8 | 0.80514 | 0.65990 |
| 6.8 kDa mitochondrial proteolipid | 0.81297 | 0.82492 |
| Cytochrome c oxidase subunit 7B, mitochondrial | 1.43398 | 1.29843 |
| Isoform 3 of Unconventional myosin-Ic | 1.90147 | 1.34096 |
| Isoform 2 of Prostaglandin-H2 D-isomerase | 1.28363 | 1.20786 |
| Tropomyosin 1, alpha, isoform CRA_j | 1.86486 | 1.55426 |
| Isoform 3 of Trinucleotide repeat-containing gene 18 protein | 1.79406 | 1.34861 |
| RNA polymerase II-associated protein 3 | 1.4116 | 1.29874 |
| Arfaptin-2 | 0.72448 | 0.69841 |
| Isoform 3 of Constitutive coactivator of PPAR-gamma-like protein 2 | 1.79406 | 1.54891 |
| UBX domain-containing protein 8 | 0.67595 | 0.57323 |
| Fission 1 (Mitochondrial outer membrane) homolog (Yeast), isoform CRA_c | 1.29885 | 1.22232 |
| CD82 antigen OS=Mus musculus | 1.34311 | 1.42548 |
| Alpha-actinin-4 (Fragment) | 1.53988 | 1.23325 |
| Solute carrier family 35 member F1 | 1.82009 | 1.21537 |
| Fanconi anemia group D2 protein homolog | 1.28886 | 1.26826 |
| Annexin (Fragment) | 1.62433 | 1.51398 |
| Acyl-CoA-binding protein | 0.71784 | 0.75489 |
| Isoform 2 of Protein lin-9 homolog | 1.36673 | 1.22617 |
| ATP synthase F(0) complex subunit C1, mitochondrial | 1.60673 | 1.24896 |
| Ashwin OS=Mus musculus | 1.53243 | 1.38784 |
| Isoform Zeta of Lamina-associated polypeptide 2, isoforms alpha/zeta | 1.34556 | 1.25647 |
| Isoform 4 of Pleckstrin homology domain-containing family B member 1 | 1.48218 | 1.35764 |
| Tropomyosin beta chain | 1.41109 | 1.26151 |
| E3 SUMO-protein ligase | 1.74983 | 1.45215 |
