# Supplementary figures and images for: Identification of learning-induced changes in protein networks in the hippocampi of a mouse model of Alzheimer's disease
Source: Transl Psychiatry. 2016 Jul 5;6(7):e849–. doi: 10.1038/tp.2016.114 (PMC4969764; doi:10.1038/tp.2016.114)

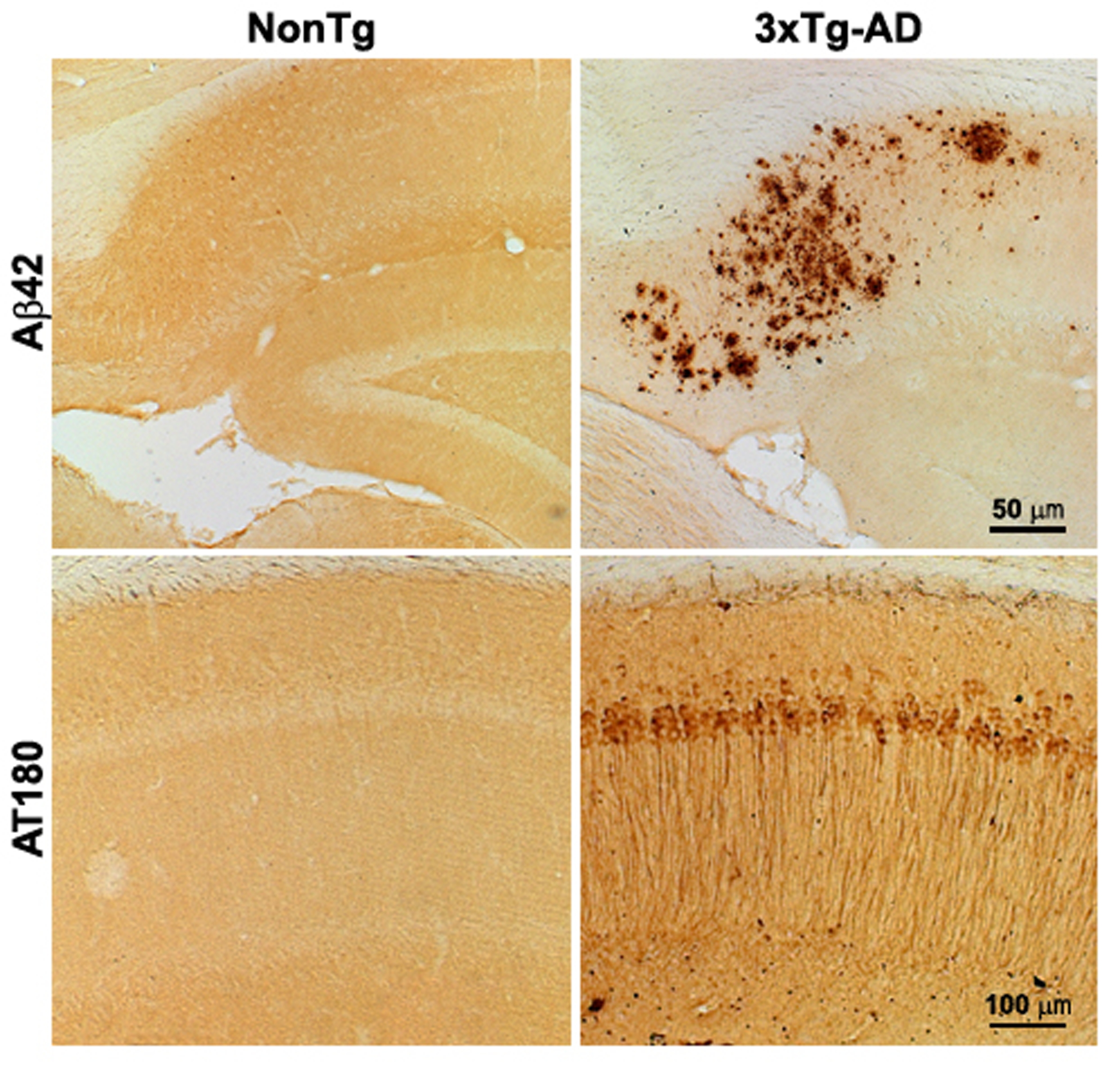

Supplement: Supplementary Figure 1 [file tp2016114x2.tif]

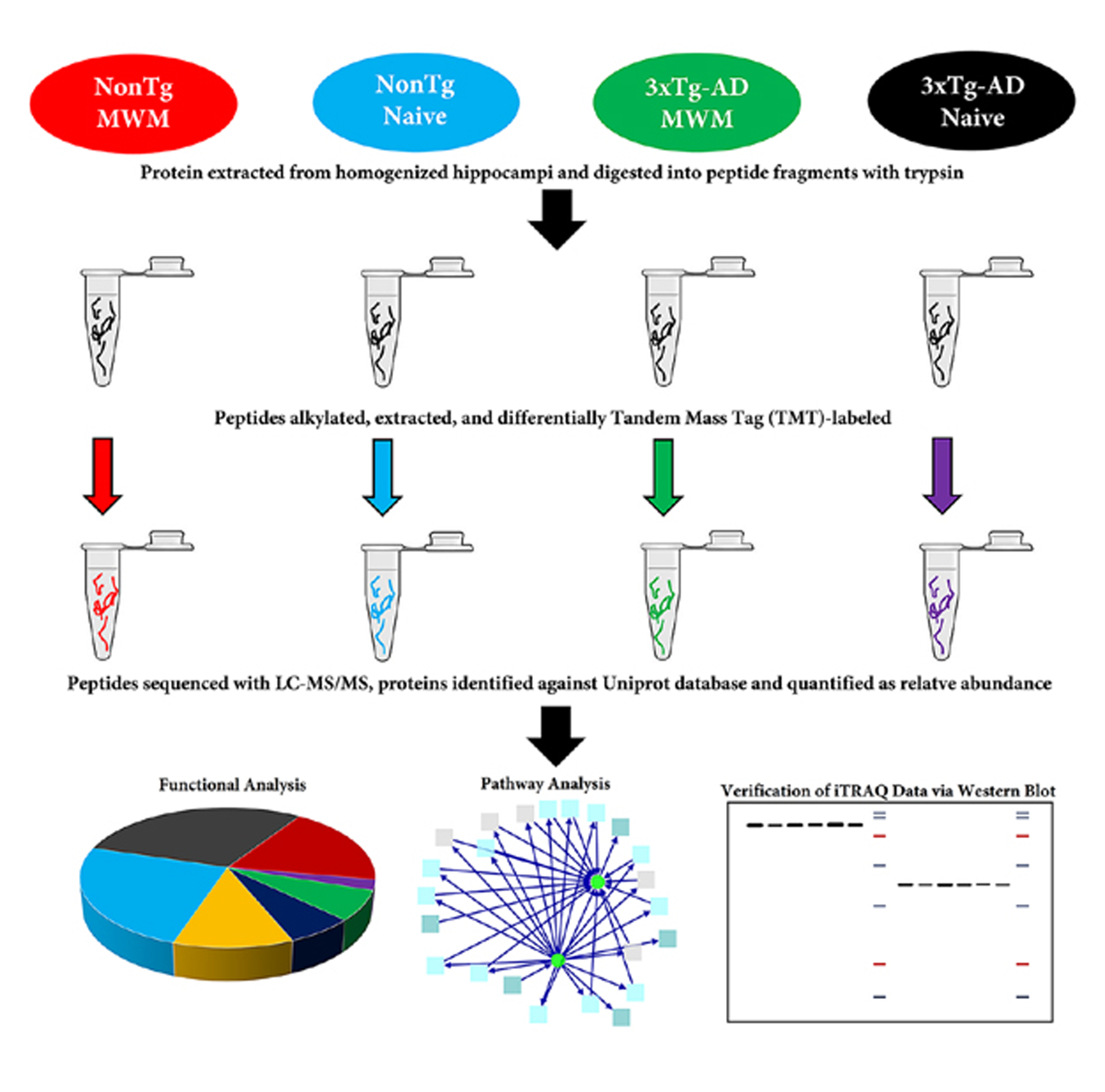

Supplement: Supplementary Figure 2 [file tp2016114x3.tif]

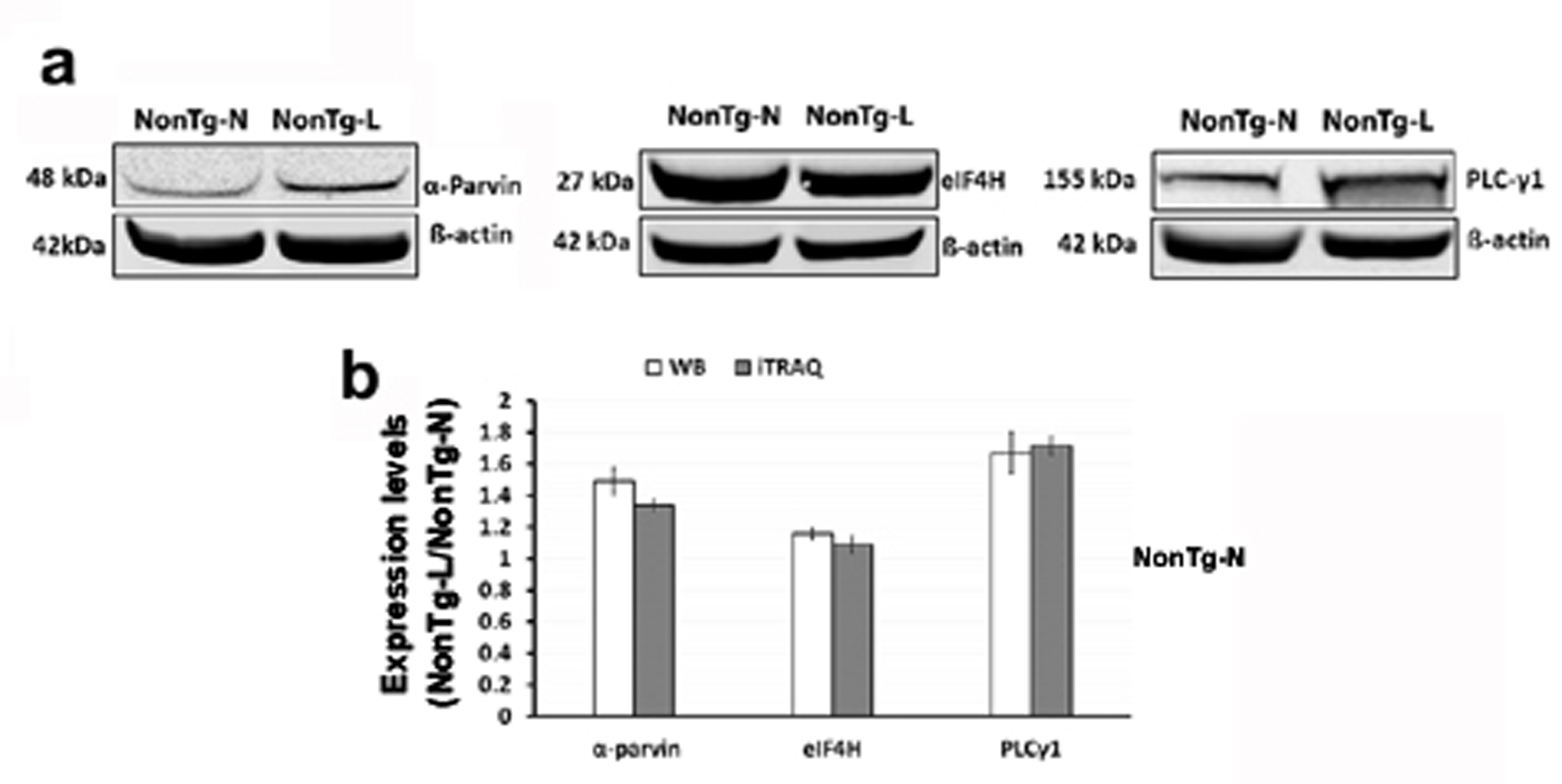

Supplement: Supplementary Figure 3 [file tp2016114x4.tif]
